# Epidemiological and Microbiological Characterization of Carbapenemase-Producing *Klebsiella pneumoniae* Isolates in a Regional Greek Hospital: A Retrospective Study

**DOI:** 10.3390/microorganisms13092132

**Published:** 2025-09-12

**Authors:** Pandora Tsolakidou, Maria Chatzidimitriou

**Affiliations:** 1 Microbiology Department, Hospital of Volos, Polymeri 134, 38222 Volos, Greece; ptsolakidou@gmail.com; 2 Department of Biomedical Sciences, Faculty of Health Sciences, International Hellenic University, 57400 Thessaloniki, Greece

**Keywords:** *Klebsiella pneumoniae* carbapenemase, New Delhi metallo-β-lactamase-1, Verona integron-encoded metallo-β-lactamase-1, antimicrobial resistance, antimicrobial stewardship program, high-risk clones, molecular epidemiology

## Abstract

Carbapenemase-producing *Klebsiella pneumoniae* (CRKP) is a critical public health threat, particularly in Greece, where high prevalence limits therapeutic options. This retrospective study analyzed 26 CRKP isolates recovered at the General Hospital of Volos between July 2024 and January 2025, aiming to correlate carbapenemase phenotypes with clinical and epidemiological parameters. Demographic, clinical, and microbiological data were extracted from patient records, and isolates underwent phenotypic carbapenemase detection, antimicrobial susceptibility testing, and molecular characterization using real-time PCR; four isolates were further analyzed using whole-genome sequencing. CRKP was detected across multiple hospital departments, notably in the Emergency Department (*n* = 5) and Intensive Care Unit (*n* = 6). KPC producers predominated (*n* = 9), followed by NDM (*n* = 6), VIM (*n* = 1), and OXA-48 (*n* = 6). All VIM- or NDM + VIM-positive cases were associated with mortality. High-risk clones, including ST15, ST11, and ST307, were identified, with one ST15 isolate harboring *bla*_NDM-1_, *bla*_VIM-1_, and chromosomal colistin resistance; this is the first such report in Greece. Colistin and gentamicin were the most active agents in vitro; three isolates were pan-drug-resistant. The findings highlight significant CRKP circulation outside ICUs, the role of horizontal gene transfer in resistance dissemination, and the need to expand screening and rapid diagnostics to non-ICU settings. Enhanced molecular surveillance targeted at infection control and strengthened antimicrobial stewardship programs are essential for limiting the spread of CRKP.

## 1. Introduction

*Klebsiella pneumoniae* (*K. pneumoniae*) is one of the most dangerous pathogenic microorganisms, responsible for severe infections in both the community and hospitals. *K. pneumoniae* belongs to the family *Enterobacterales*, and its ability to adapt and thrive in several settings is partially due to its ability to acquire resistance genes through plasmids. Its ability to transfer resistance genes to other *Enterobacterales* is alarming. It causes urinary tract, respiratory, and bloodstream infections, with particularly high mortality and frequent complications that can lead to disability. Globally, infections caused by *K. pneumoniae* are responsible for thousands of deaths annually, with economic consequences including prolonged hospitalization, high treatment costs, and reduced productivity due to extended recovery or disability [1]. *K. pneumoniae* is one of the leading bacterial causes of lower respiratory infections (LRIs). In 2021, it accounted for an estimated 176,000 LRI-related deaths, ranking third among bacterial causes after *Streptococcus pneumoniae* (505,000 deaths) and *Staphylococcus aureus* (424,000 deaths) [2]. There is a prevailing perception that antibiotic-susceptible strains of *K. pneumoniae* are mainly associated with less aggressive infections that can be treated with common antibiotics, while multidrug-resistant strains, especially those resistant to carbapenems, cause more serious and difficult-to-treat infections, particularly in patients with prolonged hospital stays or those in Intensive Care Units (ICUs) [3]. Community-acquired infections often affect patients with weakened immune systems, while hospital-acquired infections are more dangerous, as multidrug-resistant strains spread easily in healthcare settings where patients are more vulnerable to severe complications [4,5].

The hypervirulent strains of *K. pneumoniae* represent a particularly dangerous subgroup of the pathogen. These have been identified in China and mainly belong to ST11, as they are capable of causing severe infections due to enhanced virulence factors. These strains carry genes such as *rmpA2*, which encode specific traits, such as the production of a hypermucoviscous phenotype that protects them from the host immune response and makes them resistant to phagocytosis [6]. In addition, hypervirulent strains secrete toxins and synthesize siderophores such as aerobactin, which promote the dissemination of infection within the body [7]. These characteristics enable them to cause severe infections, even in previously healthy individuals in the community. Infections caused by such strains are often accompanied by increased mortality, especially when antibiotic resistance is also present, further complicating treatment and significantly impacting public health [8].

Carbapenemase-producing *K. pneumoniae* (CRKP) is a globally recognized public health challenge, especially in healthcare settings. A recent meta-analysis conducted by Lin XC et al. [9] in 2023 retained 61 articles across 14 countries and territories, estimating the global prevalence of CRKP among patients, with *K. pneumoniae* infections exhibiting a prevalence of 28.69%. South Asia had the highest prevalence at 66.04%. At the country level, Greece showed the highest prevalence worldwide at 70.61% [9]. CRKP exhibits resistance to most β-lactam antibiotics, spreads rapidly in hospitals, and is associated with significant morbidity and mortality. CRKP has emerged as a critical threat due to its resistance to most β-lactam antibiotics, rapid hospital transmission, and high mortality. The most common carbapenem-resistant strains worldwide include ST258, ST512, ST147, ST307, and ST11 [10].

Genotypic analysis of *K. pneumoniae* using next-generation sequencing (NGS) reveals the presence of genes involved in antibiotic resistance, toxin production, and the ability to form biofilms. However, the phenotypic expression of the infection depends on multiple factors, such as the site of infection, the host’s immune status, and underlying conditions such as diabetes or respiratory failure, which may increase host vulnerability [11]. The ability of *K. pneumoniae* to form biofilms is critical in chronic infections, as biofilms protect the bacteria from antibiotics and immune responses, making treatment more difficult.

*K. pneumoniae* is one of the most significant pathogens responsible for serious hospital-acquired infections in Greece, especially in Intensive Care Units (ICUs) [12]. Greece has long been a high-prevalence region, with a shifting epidemiological pattern from VIM-dominant strains to an increasing number of reports of KPC, NDM, and OXA-48 producers [13]. According to the Ambler molecular classification, carbapenemases are divided into classes A, B, and D. Class A enzymes include serine β-lactamases such as KPC (*Klebsiella pneumoniae* carbapenemase, class A serine β-lactamase); class B enzymes include metallo-β-lactamases such as VIM (Verona integron-encoded metallo-β-lactamase), and NDM (New Delhi metallo-β-lactamase); and class D enzymes include oxacillinases such as OXA-48 (oxacillinase-48) [14]. Among these, KPC-2, VIM-1, NDM-1, and OXA-48 are the most prevalent carbapenemases in Greece. The alarming rise in multidrug-resistant strains, particularly those resistant to carbapenems, limits treatment options and increases patient mortality [15]. At the same time, our understanding of the relationship between antibiotic resistance and the virulence factors that contribute to strain survival and spread remains limited.

The concentration of carbapenem-resistant *K. pneumoniae* strains in Greece, despite being a small country, is due to various factors. The excessive and inappropriate use of antibiotics, both in hospitals and in the community, promotes the emergence of resistant microorganisms. The lack of strict monitoring and control of antibiotic use, combined with inadequate adherence to hygiene protocols in hospitals, facilitates the spread of these strains [16]. Moreover, ICUs face high rates of infections from multidrug-resistant bacteria, while Greece also serves as a transit hub for travelers and migrants, aiding the importation of resistant strains from other parts of the world [17]. Finally, resistant bacteria have the ability to exchange resistance genes through genetic elements such as plasmids, accelerating the spread of resistance. All these factors contribute to the accumulation of multidrug-resistant strains in Greece.

While Intensive Care Units (ICUs) are traditionally seen as CRKP hotspots, less attention has been paid to the microorganism’s presence in general wards and Emergency Departments (EDs). Prior studies conducted in our hospital have identified high-risk clones such as ST15 and ST11, including dual-carbapenemase producers such as KPC-2 and VIM-1, NDM-1 and VIM-1, and NDM-1 and OXA-48, particularly in the medical wards of the hospital, such as the ICU [18,19,20,21,22]. In this study, we attempted to correlate the carbapenemase phenotypes with patient characteristics and hospital location.

## 2. Materials and Methods

We performed a retrospective study of CRKP isolates identified at the Microbiology Department of the General Hospital of Volos between July 2024 and January 2025. Real-Time Polymerase Chain Reaction (RT-PCR) was performed at the Laboratory of Biomedical Sciences, International Hellenic University (Table 1). Data were extracted from the Hospital’s laboratory records and patient charts. Variables included sample type, age, sex, department, carbapenemase phenotype, infection status vs. colonization status, comorbidities, antimicrobial susceptibility, and outcome (Table 2, Table 3 and Table 4).

### 2.1. Isolation, Identification, Susceptibility Testing, and Phenotypic Carbapenemase Detection

The 26 samples used in this study consisted of blood cultures, urine, and bronchial secretions. Blood cultures were incubated in a BacT/Alert^®^ automated system (bioMérieux, France). The other samples were cultured on solid plates (Bioprepare, Keratea, Greece). After the incubation, positive blood cultures were cultured on solid media.

Identification and susceptibility testing of the isolated strains were performed using an automated Vitek-2 system (Biomerieux, Marcy-l’Étoile, France). The antibiotics tested using the AST-N438 and AST-XN26 cards included amikacin, gentamicin, tobramycin, ciprofloxacin, levofloxacin, piperacillin/tazobactam, ceftazidime, ceftazidime/avibactam, cefepime, imipenem, imipenem/relebactam, meropenem, meropenem/vaborbactam, ertapenem, tigecycline, colistin, and trimethoprim–sulfamethoxazole. In addition, confirmatory tests for tigecycline and colistin were performed. For susceptibility to tigecycline, an E test (Biofilchem, Roseto, Italy) was applied. For colistin, a cation-adjusted broth microdilution method was applied (Biofilchem, Roseto, Italy). EUCAST breakpoints were applied for the interpretation of the results (https://www.eucast.org/clinical_breakpoints, accessed on 9 January 2024).

For the identification of carbapenemases, the immunochromatography method was used (NG-Carba 5, Biotech, Guipry-Messac, France).

### 2.2. Molecular Methods

Next-generation sequencing (NGS) was performed for 4 isolates (1, 2, 4, and 5) in a private laboratory (Cemia, Larisa, Greece). NGS was performed only on these four isolates (IDs 1, 2, 4, and 5) because sequencing was self-funded, and they were selected to represent different carbapenemase phenotypes and hospital departments. Total DNA extract was determined using a tissue kit (Qiagen, Hilder, Germany), and the samples were sequenced on the Ion Torrent platform. Genome assembly was carried out using Spades v 3.15.2 (https://ablab.github.io/spades/, accessed on 20 November 2024). Multilocus sequence typing was determined by comparing the genomes with the PubMLST website (https://pubmlst.org/, accessed on 20 November 2024), while serotype identification was performed using Kaptive v.1.3.0 (https://github.com/klebgenomics/kaptive, accessed on 20 November 2024). Plasmid replicon types were determined using PlasmidFinder v.2.1 (https://cge.food.dtu.dk/services/PlasmidFinder, accessed on 20 November 2024). Genes correlated with antibiotic resistance and point mutations in the assemblies were detected using AMRFinderPlus version 3.11.11 with database version 2023-04-17.1 to identify antimicrobial resistance genes (https://github.com/ncbi/amr, accessed on 20 November 2024).

Real-Time Polymerase Chain Reaction (RT-PCR) was performed at the Laboratory of Biomedical Sciences, International Hellenic University, for 15 isolates: 7, 9, 10, 12, 13, 14, 16, 18, 19, 20, 22, 23, 24, 25, and 26.

The isolation of DNA was performed from bacterial colonies grown on a plate after culture. For the extraction of DNA, the PureLink™ Genomic DNA Mini Kit (Invitrogen, ThermoFischer, Scientific, Monza, Italy) was used according to the manufacturer’s instructions. The DNA extract was stored at −20 °C until its use.

The detection of *bla*_KPC_, *bla*_NDM_, *bla*_VIM_, and *bla*_OXA-48_ was conducted using the QuantStudio™ 5 Real-Time PCR System (Applied Biosystems, ThermoFischer, Scientific, Monza, Italy). Primer and probe sequences were based on steps outlined by Ellington et al. (2016, PMID: 26795023) [23] (Table 1). PCR reactions were performed using Platinum™ qPCR SuperMix-UDG (100 reactions; Cat. No. 1730017; Life Technologies, Waltham, MA, USA) and primers synthesized at high purity (40 nmol; Biolegio, Nijmegen, The Netherlands). The thermal cycling protocol consisted of uracil-N-glycosylase (UNG) activation at 50 °C for 2 min and denaturation at 90 °C for 2 min, followed by 40 cycles of 95 °C for 15 s and 60 °C for 1 min. The results were analyzed using QuantStudio™ Design and Analysis software (Thermo Fisher Scientific, Waltham, MA, USA).

Reagents and Probes

1. Platinum qPCR SuperMix-UDG (100 reactions) (Life Technologies, USA).
2. Primer high-purity synthesis (40 nmol) (Biolegio, The Netherlands).
3. Primer purified with HPLC (40 nmol) (Biolegio, The Netherlands).
4. Probe for VIM: TDP, 3′BHQ-5′ Cys, 200 nmol (Biolegio, The Netherlands).
5. Probe for OXA-48: TDP, 3′BHQ-5′ Joe, 200 nmol (compatible with ABI 17500) (Biolegio, The Netherlands).
6. Probe for KPC: TDP, 3′BHQ-5′ Rox, 200 nmol, (Biolegio, The Netherlands).
7. Probe for NDM: TDP, 3′ΒHQ-5′ TAMRA, 200 nmol, (Biolegio, The Netherlands) (Table 3).

## 3. Results

### 3.1. Clinical Characteristics, Antibiotic Susceptibility, and Carbapenemase Phenotypes

In total, 26 patients were included (13 males and 13 females), with a median age of 77 years (range: 21–93). The majority of isolates were from urine (*n* = 21), followed by blood and bronchial secretions. The median age of patients with CRKP isolation was 76 years, with the majority of cases occurring in individuals over 70. CRKP was identified across multiple hospital departments, with the highest number of isolates originating from the Intensive Care Unit (*n* = 6), the Second Internal Medicine Department (*n* = 5), and the Emergency Department (ED) (*n* = 5). Among the 26 cases, 14 (53%) were classified as infections and 12 (46%) were classified as colonization (Table 2). Frequent comorbidities included cancer (*n* = 9), mechanical ventilation (*n* = 4), heart failure (*n* = 4), and hypertension (*n* = 4). The median Charlson Comorbidity Index (CCI)—based on the number of underlying conditions reported for the listed patients—was 3 (Table 3). Notably, mortality was observed in three infected and one colonized patient. KPC producers were the most frequent phenotype (*n* = 10), followed by NDM + OXA-48 (*n* = 6), NDM alone (*n* = 4), and dual or multiple carbapenemase combinations, including NDM + KPC and NDM + VIM. While most patients with KPC-producing strains showed clinical improvement, all isolates harboring VIM or NDM+VIM were associated with fatal outcomes. A high proportion of isolates, regardless of infection status, were found in patients with multiple comorbidities, including bladder cancer and heart failure (Table 2 and Table 3).

Colistin (*n* = 14) and gentamicin (*n* = 10) were the most effective in vitro antibiotics. Three isolates were pan-drug-resistant (PDR). All KPC isolates were susceptible to ceftazidime/avibactam, imipenem/relebactam, and meropenem/vabobarctam (Table 4).

Detected phenotypes included NDM (*n* = 6), KPC (*n* = 9), VIM (*n* = 1), and OXA-48 (*n* = 6), with multiple co-productions noted: NDM + OXA-48 (n = 6), NDM + KPC (*n* = 3), KPC + VIM (*n* = 1), and NDM + VIM (*n* = 1) (Table 2).

### 3.2. Molecular Analysis

NGS revealed diverse resistance determinants and mobile genetic elements (Table 5). The most common antimicrobial resistance genes detected for β-lactams included *bla*_NDM_, *bla*_KPC-2_, *bla*_VIM_, and *bla*_CTX-M15_. The STs included ST11, ST15, and ST307. The most common replicons were IncF incompatibility. No aerobactin operon was found in the genomes. The capsular types were designated as KL-14, KL-48, and KL-102. Mutations to the *pmrB* gene were detected [18,19,20,21].

Real-time PCR supported rapid phenotype confirmation.

## 4. Discussion

Our findings demonstrate the phenotypic and genotypic diversity of CRKP in a regional hospital setting. Notably, CRKP was frequently isolated in non-ICU environments, particularly in the ED, suggesting potential for undetected transmission. The coexistence of multiple carbapenemases within isolates highlights the role of horizontal gene transfer under antimicrobial pressure.

The distribution of CRKP across both critical and non-critical care units, particularly the Emergency Department and Internal Medicine, highlights a broader epidemiological challenge beyond the ICU setting. The identification of CRKP in the Emergency Department suggests possible healthcare-associated transmission from prior hospital stays or nursing home residents [24]. The coexistence of multiple carbapenemases reflects ongoing gene exchange and selective pressure [25]. Molecular surveillance (e.g., NGS) can enhance infection control and prevention. These findings suggest that screening strategies should be reconsidered to include patients admitted through the ED and medical wards, especially those with recent healthcare contact or advanced age. Methods such as FilmArray or real-time PCR could enhance timely intervention in bloodstream infections due to carbapenemase-producing strains. The cost and difficulty of implementing these methods in laboratory routine are prohibitive in many Greek hospitals. Although carbapenemase type appeared to influence clinical outcomes—particularly in cases involving VIM or dual carbapenemases—our data suggest that age and comorbidities were equally, if not more, significant predictors of adverse outcomes [26].

The inappropriate and excessive use of antibiotics, both in community and healthcare settings, represents a major driver of antimicrobial resistance [27]. Empirical administration without microbiological confirmation, suboptimal dosing or treatment duration, and the indiscriminate use of broad-spectrum agents exert substantial selective pressure on bacterial populations, promoting the emergence and dominance of resistant strains. This phenomenon, combined with the efficient horizontal transfer of resistance genes among bacteria, significantly reduces available therapeutic options and contributes to increased morbidity and mortality from infections that were once readily treatable. Conversely, the lack of availability of antibiotics active against metallo-β-lactamase–producing strains, such as aztreonam/avibactam and cefiderocol, significantly restricts therapeutic options in Greek hospitals [28]. In the General Hospital of Volos, the Antimicrobial Stewardship Committee lacks the capacity to effectively monitor antibiotic use due to a serious lack of personnel. Clinicians are often reluctant to modify a broad-spectrum regimen that appears clinically effective, even when susceptibility testing indicates sensitivity to narrower-spectrum alternatives, thereby perpetuating unnecessary selective pressure [29].

Another major barrier is the limited availability of effective therapeutic options and the absence of internal laboratory information systems (LIS), which prevents the timely notification of treating physicians. A fatal example is that of the patient with ID 2 who was transferred to the ICU of the General Hospital of Volos and died of septic shock due to polymicrobial bacteremia caused by *Enterobacter cloacae* (VIM-producer), *K. pneumoniae*, and vancomycin-resistant *Enterococcus faecium* (VRE) [18]. Next-generation sequencing (NGS) revealed that the *K. pneumoniae* isolate belonged to the high-risk clone ST15 and co-produced the NDM-1 and VIM-1 metallo-β-lactamases. In addition to extensive β-lactam resistance, the strain exhibited chromosomally encoded resistance to colistin—a rare but highly stable resistance mechanism—rendering all β-lactams, including carbapenems and extended-spectrum cephalosporins, ineffective, along with most second-line options. Moreover, the isolate carried *bla*_CTX-M-14_, aminoglycoside, and trimethoprim resistance genes within a class 1 integron, as well as an efflux pump gene, further reinforcing its multidrug-resistant profile. The therapeutic implications were grave, as the only rational treatment—aztreonam/avibactam—is not available in Greece due to regulatory restrictions; even aztreonam alone, which might have offered partial coverage, is not circulated nationally because of pharmaceutical reluctance to support its distribution.

This constellation of resistance determinants in a globally disseminated clone such as ST15 underscores a public health emergency occurring in slow motion. It was the first documented case of ST15 harboring both NDM-1 and VIM-1, along with colistin resistance in Greece [18]. As such, it signals a dangerous ongoing convergence of resistance genes into successful lineages capable of widespread healthcare-associated transmission. The occurrence of death in colonized patients further emphasizes that colonization by CRKP is not clinically benign in vulnerable hosts. The favorable response observed in most KPC-related cases likely reflects the effective use of ceftazidime/avibactam, while therapeutic limitations remain for NDM and VIM producers. Taken together, these observations underscore the need for comprehensive patient-centered risk stratification in managing CRKP, beyond the molecular profile of the isolate.

The predominance of NDM and KPC phenotypes, in line with national trends, underscores the need for robust antimicrobial stewardship and real-time molecular diagnostics [22]. The detection of high-risk clones (e.g., ST15, ST11) suggests concurrent clonal expansion and horizontal gene dissemination. This finding contrasts with the national epidemiology, where ST258 predominates, underscoring the importance of generating local epidemiological data [12]. Early detection using RT-PCR and confirmatory NGS is critical for guiding therapy and preventing outbreaks.

### Limitations of This Study

This study has several limitations. Data on hospitalizations within the previous six months and antibiotic use within the preceding three months were not available. The sample size was small, and neither multilocus sequence typing nor pulsed-field gel electrophoresis could be performed on all 26 isolates due to a lack of appropriate laboratory equipment. Furthermore, next-generation sequencing (NGS) was conducted only on four isolates, as the project relied solely on self-funding.

## 5. Conclusions

CRKP in this regional hospital shows diverse resistance profiles, with a notable prevalence in the Emergency Department. Ongoing molecular characterization, rapid diagnostics, and infection control programs are critical for mitigating the spread of the microorganism and improving patient outcomes.

## Figures and Tables

**Table 1:** Primers and probes for real-time PCR. Definitions: All primers were used at 40 nmol concentration per reaction. Fluorophores: ROX, TAM (TAMRA), JOE, CY5, Cy5.5. BHQ: Black Hole Quencher (BHQ-1, BHQ-2, and BHQ-3) used according to probe compatibility.

Gene	Type	Name	Sequence (5′ → 3′)	5′ Label	3′ Label
KPC	Primer (forward)	KPC_fwd	GCAGCGGCAGCAGTTTGTTGATT	–	–
	Primer (reverse)	KPC_rev	GTAGACGGCCAACACAATAGGTGC	–	–
	Probe	KPC_probe	CAGTCGGAGACAAAACCGGAACCTGC	ROX	BHQ-2
NDM	Primer (forward)	NDM_fwd	CCAGCAAATGGAAACTGGCGAC	–	–
	Primer (reverse)	NDM_rev	ATCCAGTTGAGGATCTGGGCG	–	–
	Probe (ABI7500)	NDM_probe_ABI7500	ACCGAATGTCTGGCAGCACACTTC	TAM	BHQ-2
OXA-48	Primer (forward)	OXA48_fwd	GATTATGGTAATGAGGACATTTCGGGC	–	–
	Primer (reverse)	OXA48_rev	CATATCCATATTCATCGCAAAAAACCACAC	–	–
	Probe (ABI7500)	OXA48_probe_ABI7500	CCATTGGCTTCGGTCAGCATGGCTTGTTT	JOE	BHQ-1
VIM	Primer (forward)	VIM_fwd	TTGCTTTTGATTGATACAGCGTGGGG	–	–
	Primer (reverse)	VIM_rev	GTACGTTGCCACCCCAGCC	–	–
	Probe	VIM_probe	TCTCGCGGAGATTGAAAAGCAAATTGGACTTCC	CY5	BHQ-3

**Table 2:** Clinical and microbiological characteristics of CRKP isolates (Abbreviations: ID = isolate identification number, ICU = Intensive Care Unit, M = Male, F = Female).

ID	Department	Sex	Age	Laboratory ID	Sample	Infection(I)/ Colonization ©	Outcome
1	ICU	M	21	838-7-24	Urine	Infection	Clinical improvement
2	ICU	M	62	A436-7-24	Blood	Infection	Death
3	2nd Internal	F	90	1135-7-24	Urine	Infection	Death
4	Urology	M	70	989-7-24	Urine	Infection	Clinical improvement
5	ICU	F		A165-8-24	Blood	Infection	Clinical improvement
6	Peritoneal dialysis	M	77	1604-8-24	Urine	Colonization	Clinical improvement
7	Urology	M	6	640-8-24	Urine	Infection	Clinical improvement
8	Anemia treatment unit	F	54	745-8-24	Urine	Colonization	Clinical improvement
9	Urology	Μ	76	1141-8-24	Urine	Colonization	Clinical improvement
10	Surgical	F	79	1219-9-24	Urine	Colonization	Clinical improvement
11	Emergency	F	93	230-9-24	Urine	Colonization	Clinical improvement
12	2nd Internal	F	89	1626-9-24	Urine	Colonization	Death
13	Emergency	M	78	249-9-24	Urine	Infection	Clinical improvement
14	ICU	M	69	274-9-24	Bronchial secretions	Colonization	Death
15	Chemotherapy	M	77	405-10-24	Urine	Colonization	Clinical improvement
16	Emergency	F	73	469-10-24	Urine	Colonization	Clinical improvement
17	Orthopedics	F	85	A5376-10-24	Blood	Infection	Death
18	ICU	M	64	1189-10-24	Bronchial secretions	Colonization	Clinical improvement
19	2nd Internal	F	85	1413-10-24	Trauma	Infection	Clinical improvement
20	ICU	M	77	1256-11-24	Bronchial secretions	Colonization	Clinical improvement
21	2nd Internal	M	60	1085-11-24	Urine	Colonization	Clinical improvement
22	1st Internal	F	84	1021-11-24	Urine	Infection	Clinical improvement
23	Emergency	F	70	1454-12-24	Urine	Infection	Clinical improvement
24	2nd Internal	F	82	1302-12-24	Urine	Infection	Clinical improvement
25	2nd Internal	F	70	389-12-24	Urine	Infection	Clinical improvement
26	Department	F	65	685-1-25	Urine	Infection	Clinical improvement

**Table 3:** Reason for admission and comorbidities (Abbreviation: ID = isolate identification number).

ID	Laboratory ID	Reason for Admission	Comorbidities—Risk Factors
1	838-7-24	road traffic accident	mechanical ventilation
2	A436-7-24	dehydration, heatstroke	severe hepatic steatosis, rhabdomyolysis, hyponatremia, respiratory collapse, mechanical ventilation, fever
3	1135-7-24	anuria, hematuria	heart failure, cerebral infarction, idiopathic hypertension
4	989-7-24	surgery	prostatic hypertrophy
5	A165-8-24	respiratory distress	renal cancer, metastatic cancer of the pleura, immunotherapy, mechanical ventilation, central venous catheterization
6	1604-8-24	routine screening	bladder cancer, peritoneal dialysis
7	640-8-24	fever	bladder cancer
8	745-8-24	routine screening	β-thalassemia, osteoporotic fractures
9	1141-8-24	fracture	bladder cancer, interstitial lung disease
10	1219-9-24	ileus	heart failure, chronic obstructive disorder (COPD), type 2 diabetes, overweight
11	230-9-24	skull bone fracture	not reported
12	1626-9-24	dysarthria, anarthria	not reported
13	249-9-24	fever	bladder cancer, liver cancer, prostatic hypertrophy, multiple myeloma, peripheral vasculopathy
14	274-9-24	pain	short bowel syndrome, mesenteric ischemia, heart failure, mechanical ventilation
15	405-10-24	not reported	colon cancer, lung cancer
16	469-10-24	not reported	bladder cancer
17	A5376-10-24	hip fracture	not reported
18	1189-10-24	dyspnea, oliguria	heart failure, alcoholic cirrhosis, coronary artery disease, atrial fibrillation, type 2 diabetes, hypertension
19	1256-11-24	road traffic accident	mechanical ventilation
20	1413-10-24	Intertrochanteric fracture of the femur	not reported
21	1085-11-24	anemia	bladder papilloma, lung cancer, thrombophilia, non-Hodgkin lymphoma
22	1021-11-24	hematuria	hepatitis virus B, patient bedridden due to a bone fracture, COPD
23	1454-12-24	fever	hip arthroplasty, hypothyroidism
24	1302-12-24	urinary infection	bladder cancer, idiopathic, hypertension, type 2 diabetes
25	389-12-24	fever	heart failure, cerebral infarction, idiopathic hypertension
26	685-1-25	lower respiratory tract infection	not reported

**Table 4:** Antibiotic susceptibility of 26 carbapenem-resistant *K. pneumoniae* isolates. (Abbreviations: CAZ-AVI = ceftazidime/avibactam; MVB = meropenem/vaborbactam; IMR = imipenem/relebactam; TMP-SMX = trimethoprim–sulfamethoxazole; PDR = pan-drug-resistant).

ID	Carbapenemase Phenotype	CAZ-AVI	MVB	IMR	Colistin	Gentamicin	Amikacin	TMP-SMX	Other Notes
1	NDM	R	R	R	R	R	R	R	PDR
2	NDM + VIM	R	R	R	R	R	R	R	PDR
3	NDM + OXA-48	R	R	R	S	S	R	R	—
4	KPC	S	S	S	S	S	R	S	—
5	NDM + OXA-48	R	R	R	S	R	R	S	—
6	NDM	R	R	R	R	R	R	R	PDR
7	NDM + KPC	R	R	R	S	R	R	R	—
8	KPC	S	S	S	S	R	R	R	—
9	NDM + KPC	R	R	R	S	S	R	R	—
10	NDM + OXA-48	R	R	R	S	R	R	S	—
11	KPC	S	S	S	S	S	R	R	—
12	NDM	R	R	R	S	R	R	R	—
13	KPC	S	S	S	S	R	R	S	—
14	KPC	S	S	S	S	S	R	R	—
15	KPC + VIM	R	R	R	S	S	R	R	—
16	NDM + OXA-48	R	R	R	S	R	R	R	—
17	VIM	R	R	R	S	S	R	R	Susceptible to aztreonam
18	KPC	S	S	S	S	S	S	R	
19	NDM	R	R	R	S	R	R	R	Susceptible to aztreonam
20	NDM	R	R	R	S	R	R	S	—
21	NDM + KPC	S	S	S	S	S	R	R	—
22	KPC	S	S	S	S	S	R	R	—
23	KPC	S	S	S	S	R	R	R	—
24	NDM + OXA-48	R	R	R	S	S	R	S	—
25	NDM + OXA-48	R	R	R	S	S	S	R	—
26	KPC	S	S	S	S	S	S	R	

**Table 5:** Comparison between four isolates with IDs 1, 2, 4, and 5.

Feature	ST11 (838Gr) I.D 1	ST15 (A436) I.D 2	ST307 (U989) I.D 4	ST11 (A165) I.D 5
Specimen	Urine	Blood	Urine	Blood
Carbapenemases	*bla*_NDM-1_	*bla*_NDM-1_, *bla*_VIM-1_	*bla*_KPC-2_	*bla*_NDM-1_, *bla*_OXA-48_
Other β-lactamases	*bla*_CTX-M-15_, *bla*_TEM-1B_, *bla*_VEB-1_, *bla*_SHV-11_, *bla*_OXA-10_	*bla*_CTX-M-15_, *bla*_SHV-28_, *bla*_TEM-1_, *bla*_OXA-1_	*bla*_CTX-M-15_, *bla*_SHV-106_, *bla*_VEB-1_, *bla*_TEM-1B_, *bla*_OXA-1_, *bla*_OXA-10_	*bla*_CTX-M-14b_, *bla*_SHV-182_
Resistance profile	Pan-drug resistant (PDR)	PDR	Susceptible to colistin, gentamicin, ceftazidime/avibactam (CAZ-AVI)	Susceptible to gentamicin, colistin, trimethoprim–sulfamethoxazole (TMP-SMX)
Replicons	IncFIA, IncC, IncR, repB	IncA/C2, IncFIB (K), IncFIA (HI1) IncFII (K)	IncFIB (pQil)/FII (K), IncA/C, ColRNAI	IncFIB (K), IncFIA (HI1), IncFII (K), IncR, Col440II
Virulence genes	*entA-S*, *iroN*, *fyuA*, *iutA*, *T6SS*	*mrk*, *fim*, *yersiniabactin* cluster	*iutA*, *fyuA*, *mrkA*, *fimH*, *clpK1*, *irp2*, *traT*	*irp1*, *irp2*, *ybtE*, *iutA*, *ompA*, *rcsA/B*
Capsular type/O-antigen	KL24/O2a	KL48	KL102/O2afg	KL24/O2a
Year of Isolation	2024	2024	2024	2024

## Data Availability

The whole genomes of *K. pneumoniae* isolates has been deposited at DDBJ/ENA/GenBank under the accession numbers PRJNA1222132, PRJNA1186229, PRJNA1150742, and PRJNA1147747.
